# Feasibility of Designing, Manufacturing and Delivering 3D Printed Ankle‐Foot Orthoses: An Updated Systematic Review

**DOI:** 10.1002/jfa2.70097

**Published:** 2025-12-09

**Authors:** Joyce Z. Wang, Elizabeth A. Wojciechowski, Thomas Paine, Joshua Burns, Tegan L. Cheng

**Affiliations:** ^1^ School of Health Sciences Faculty of Medicine and Health & Children's Hospital at Westmead University of Sydney Sydney Australia; ^2^ EPIC Lab The Children's Hospital at Westmead Sydney Australia; ^3^ Paediatric Gait Analysis Service of NSW The Children's Hospital at Westmead Westmead Australia; ^4^ Department of Orthotics The Children's Hospital at Westmead Sydney Australia; ^5^ Department of Epidemiology and Cancer Control St. Jude Children's Research Hospital Memphis Tennessee USA

**Keywords:** 3D printing, additive manufacturing, AFO, ankle foot orthoses, gait

## Abstract

**Background:**

Ankle‐foot orthoses (AFOs) are commonly prescribed to manage lower limb impairments, especially foot drop in neurological disorders. With the evolution of 3D technology, digital acquisition using 3D scanning and modelling using computer aided design software is becoming more commonplace to produce AFOs. Our previous systematic review in 2019 identified an emerging field of 3D printed AFOs and we highlighted biomechanical effects, mechanical properties and self‐reported outcomes such as comfort. To cover the rapidly growing literature on the effects of 3D printed AFOs in clinical populations, the aim of this systematic review was to update an earlier review from 2019 to determine the feasibility and effect of 3D printed AFOs on biomechanical and satisfaction outcomes.

**Method:**

Seven electronic databases were searched from 1985 to July 2025. Original research papers of any design from healthy or clinical populations of any age were eligible for inclusion. Studies must have investigated the effect of 3D printed AFOs in healthy or pathological populations. The quality of the evidence was assessed using QualSyst.

**Results:**

Twenty‐eight papers were included in the updated systematic review. The use of 3D printing methods and materials varied markedly, where fused deposition modelling was more prevalent in recent literature than selective laser sintering, and Nylon 12 was most tested. The sample sizes were all smaller than 12. Walking speed and step length of people wearing 3D printed AFOs were mostly improved compared to those with other AFOs and shod or barefoot only. 3D printed AFOs generally had similar or higher satisfaction scores than traditional AFOs. Although levels of evidence were all lower than four, 10 papers had excellent study quality.

**Conclusion:**

The use of additive manufacturing in AFO fabrication has rapidly increased in the past few years. The novel designed 3D printed AFOs might have potential benefits over traditional designs in terms of biomechanical outcomes. 3D printed AFOs have been further proven to be comparable to traditional ones. Further research is encouraged to conduct with more specific condition characteristics such as cerebral palsy within a specific GMFCS level, longitudinal clinical trials and testing in a home or natural environment. Establishing a standard for AFO evaluation and reporting is also recommended.

## 1 Introduction

Custom made, prefabricated and customised ankle‐foot orthoses (AFOs) are commonly applied orthotic interventions to support people with conditions such as neuromuscular dysfunction for improving walking kinematics, supporting daily function and accommodating foot deformities [1]. Traditional customised AFOs have typically been manufactured utilising thermoplastic materials after modification to a plaster positive model cast from a plaster of Paris cast. The process takes several hours of hands‐on labour time to prepare and mould, requires significant skill to apply and the casts can be easily deformed [1, 2]. The complete fabrication and manufacturing process typically takes approximately 6–8 h per AFO and needs to be staged over a few days as the thermoplastic AFO needs to be completely cooled down before an orthotic clinician or technician can cut it off. There are also health and safety concerns with the traditional manufacturing method.

As the technology rapidly develops and widely spreads, additive manufacturing has evolved in terms of speed, material variety and applications across different industries. The utilisation of the 3D technologies is becoming common in the orthotics industry for replacing traditional plaster room workshops as access to 3D printers continues to improve and production costs are decreasing [3, 4]. Although a randomised double‐blind trial did not find significant improvements within the time to delivery comparing traditional and additive manufacturing methods [2], 3D printing combined with 3D scanning and computer aided design (CAD) brings possibilities for more novel designs and lighter designs with the same strength characteristics as their traditional thermoplastic AFO counterparts. Advanced 3D printing techniques such as printing with varying materials and thicknesses have also been explored in the prosthetics and orthotics field to achieve a better mechanical performance of the orthotics to enhance strength and flexibility of the device, without compromising patient safety or patient outcomes [5]. Moreover, compared to plaster casting technique, additive manufacturing could reduce physical exertion, repetitive manual tasks, vibration from power tools and exposure to harmful fumes, radiant heat and loud noises. The environmental impact can also be reduced by scaling back or eliminating complex supply chains and reduce waste and emissions associated with traditional manufacturing processes to help promote a more sustainable work process.

Despite its increasing use, there is still a dearth of research on the clinical outcomes of 3D printed AFOs. Our previous systematic review from 2019 highlighted that the biomechanical effects of 3D printed AFOs were comparable to traditionally manufactured AFOs, but only 11 studies were included [6]. Since a rapid increase in interest and study in the space, an update of the previous review is necessary. The specific goal of this systematic review is to investigate the feasibility and effectiveness of designing and manufacturing 3D printing AFOs by evaluating factors such as biomechanical effects and user satisfaction in clinical and healthy populations. This current systematic review differs from the previous one as it does not include mechanical testing, rather placing a greater focus on clinical outcomes.

## 2 Methods

This systematic review was conducted by following the Preferred Reporting Items for Systematic Reviews and Meta Analysis (PRISMA) 2020 statement. The PRISMA checklist can be found in Table S1. This systematic review is an update of the paper published in 2019 [6] and the approach for this current systematic review was largely the same as the previous one; however, the mechanical testing focus has been removed, and search strategies have been refined. Prior to conducting the searches, the protocol was developed, and the study was registered and updated in PROSPERO (CRD42024566633). Although the PROSPERO protocol was registered using the methodology of the prior systematic review, several modifications were made to strengthen the search terms, generate deeper insights into the use of 3D printed AFOs in functional tests and provide more relevant quality assessments. These changes were made prior to the screening phase of the current study. For example, to broaden the evidence base of the functional impacts of 3D printed AFOs, both clinical and healthy populations were included. As this review focused on clinical outcomes, the range of comparators was broadened to include both conventionally fabricated AFOs and non‐AFO conditions.

### 2.1 Search Strategy and Eligibility Criteria

Electronic database searches were performed in MEDLINE (via OvidSP), EMBASE, AMED (via OvidSP), CINAHL (via EBSCO), Web of Science, Scopus, Cochrane and ProQuest Central according to search terms related to 3D printing combined with terms related to AFOs while excluding nonclinical studies. The search strategy was developed for MEDLINE and adapted for use in other databases and the search strategy can be found in Supporting Information S1: Additional file 2. To align with the earlier review, the search covered articles published until 28th of July 2025. Hand searches were also performed to capture publications such as the citations of included papers.

Clinical studies written in English of any sample size investigating the effects of 3D printed AFOs were eligible. Clinical studies written in English of any sample size investigating the effects of 3D printed AFOs were eligible. There were no limits of age, gender or ethnicity. The participants could be either healthy or from clinical populations. Compared to the original systematic review, the inclusion criteria were modified to only include studies with functional tests on people. All journal articles were eligible and there were no limits on 3D printing technology. However, review articles, conference proceedings, book chapters and theses were excluded. Computer‐aided manufacturing techniques such as carving were excluded and studies investigating AFOs of which the main section was not 3D printed were excluded. If there were uncertainties about the manufacturing methods, authors were contacted.

### 2.2 Study Selection, Data Extraction and Quality Assessment

The title/abstract screening, full‐text screening processes, data extraction and quality assessment were all conducted using Covidence (Covidence systematic review software, Veritas Health Innovation, Melbourne, Australia). Duplicates were automatically and manually removed. Two reviewers (JZW and TLC) performed title and full‐text screening, data extraction and quality assessment independently. Disagreements were first resolved by the two reviewers; however, if consensus was not achieved, a third reviewer (EW) was consulted. Consensus was achieved for all included studies.

Data extraction included type of study design, participant characteristics, details of manufacturing methods and outcome measurements with reported results. The Oxford Centre for Evidence‐Based Medicine 2011 Levels of Evidence (OCEBM Levels) was used to determine a level of evidence for each study based on the design [7]. The QualSyst tool which is designed for assessing the quality of quantitative studies was used to assess the overall quality of each included study [8]. The checklist includes 14 items which scores from zero (No) to two (Yes) and a maximum total score of 28. The quality of studies with an overall score over 85% was identified as excellent, 75%–85% as good, 55%–75% as adequate and less than 55% as limited [9]. Assessment with the American Academy for Cerebral Palsy and Developmental Medicine (AACPDM) was not used in this review. Meta‐analysis was not applicable for this review due to the clinical and methodological heterogeneity of the included papers.

## 3 Results

### 3.1 Description of Included Studies

**FIGURE 1:**
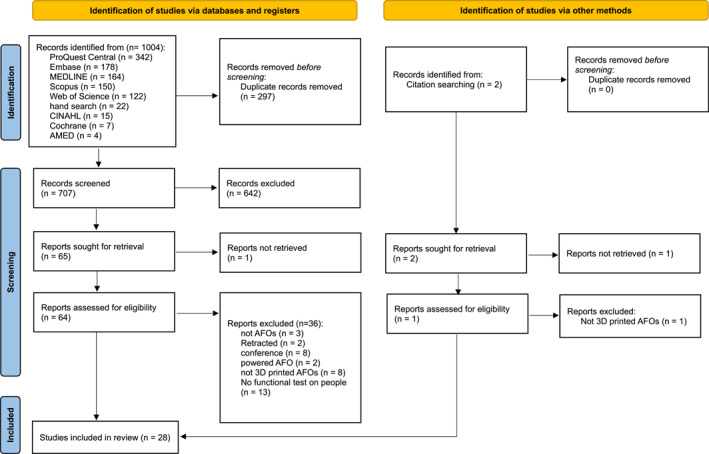
PRISMA flow diagram for the selection process of the systematic review.

In total, 1004 articles were identified from the initial database searches from which 297 duplicates were removed. The remaining 707 articles were screened by the abstract and title which left 64 for full text screening. Hand searching was performed and identified additional 22 publications. The whole selection process can be seen in Figure 1. A total of 28 studies were included and underwent quality assessment of which the study designs were mostly case control (71.4%) [10, 11, 12, 13, 14, 15, 16, 17, 18, 19, 20, 21, 22, 23, 24, 25, 26, 27, 28, 29]. Twenty studies were rated as OCEBM Level 4 evidence [11, 13, 14, 15, 16, 17, 18, 19, 20, 21, 22, 23, 24, 25, 27, 28, 29, 30, 31, 32, 33], whereas eight were lower (Level 5) [10, 12, 26, 33, 34, 35, 36, 37]. Sample sizes of the studies were all less than 12, where eight studies (28.6%) included a single participant [10, 14, 16, 26, 28, 30, 31, 37]. The most common conditions were foot drop (32.1%), followed by post stroke (25.0%) and CMT (7.1%). Eight studies recruited only healthy participants (28.6%) [12, 26, 31, 32, 33, 35, 36, 37].

### 3.2 3D Printing Methods and Materials

Eleven papers (39.3%) utilised the fused deposition modelling (FDM) method as the 3D printing method [10, 11, 16, 17, 20, 22, 23, 29, 30, 31, 32], which was the same as selective laser sintering (SLS) [12, 13, 14, 15, 18, 19, 27, 33, 34, 36, 37], followed by SLA (stereolithography) [24, 26]. The material types used for manufacturing AFOs were varied including Nylon 12 (PA12) [18, 19, 25, 27, 28, 29, 31, 37], polylactide (PLA) [10, 20, 23], fibreglass reinforced polyamide [14, 15], polycarbonate [11, 21], acrylonitrile butadiene styrene (ABS) [22], polyurethane [30], polyester elastomer [17], Nylon composite carbon fibre [22], carbon fibre [21], Accura 40 resin and DSM Somos 9120 Epoxy Photopolymer [26], DuraForm EX [12] and Somos NeXt [24]. However, two studies did not specify the printing materials [34, 35] and three did not mention the printing methods [21, 28, 35]. 60.1% of the included study 3D printed unique variations of passive dynamic AFOs [11, 12, 13, 14, 15, 18, 19, 21, 22, 23, 26, 27, 28, 31, 35, 36, 37], while three were hinged AFOs [20, 29, 32] and two printed solid AFOs [26, 29]. Eight studies (28.6%) did not clearly define details of the 3D printed AFOs [10, 17, 24, 25, 27, 30, 33, 34]. In terms of outcome measurements, most of the included studies (71.4%) assessed walking ability using 3D gait analysis [11, 12, 13, 14, 15, 16, 17, 18, 21, 22, 23, 24, 25, 26, 27, 28, 29, 32, 34, 37], and 11 studies evaluated aspects of user satisfaction or perceived comfort [14, 15, 16, 17, 20, 23, 24, 29, 30, 31, 33]. Further details can be found in Table 1.

**TABLE 1:** Results and study characteristics of the included studies.

	Participants' characteristics		Intervention			Outcomes and results		
Reference	Sample size	Condition	3D printing AFO type	3D printing method and material	Control AFO type	Outcomes	Authors' results	OCEBM level
Abdalsadah 2021 [10]	1	Foot drop (M, 22 years)	NR	FDM	Traditionally made AFO	Vibration test Gait analysis (observation)	3DP AFOs had less vibration than traditional AFOs.	5
				PLA				
Arch 2015 [12]	2	Healthy (1M, 1F, 24 and 25 years)	PDAFO	FDM	Shod only	Temporal spatial kinematics and kinetics: 3DGA	Average peak net plantar flexion moments for all PDAFO conditions were within 8.3% and 11.6% of the no PDAFO conditions Peak ankle plantar flexion moments remained unchanged across PDAFO stiffness. Decreased ankle dorsiflexion during midstance in all PDAFO conditions, whereas natural dorsiflexion excursions were reached with no PDAFO.	5
				DuraForm EX				
Arch 2016 [11]	2	Post stroke (1M, 1F, 43 and 49 years)	PDAFO	FDM	Traditionally made AFO; No AFO	Temporal spatial, kinematics and kinetics: 3DGA	Dorsiflexion decreased with PDAFO compared with the baseline conditions and was lower than average excursions for speed‐matched normal individual. PDAFO increased the subjects' net peak plantarflexion moment. PDAFO improved both subjects' paretic knee and hip joint kinematics during mid‐ and late stance but excessively reduced dorsiflexion.	4
				Medical grade polycarbonate (PC ISO)				
Banga 2022 [34]	10	Foot drop	NR	SLS	NR	Temporal spatial, kinematics and kinetics: 3DGA	3DP AFOs enhanced 75% human gait of foot drop patients.	5^a^
				NR				
Caravaggi 2022 [15]	1	Foot drop due to paraparesis in severe discarthrosis (M, 67 years)	PDAFO	SLS	Shod only; Off‐the‐shelf leaf spring AFO (Codivilla)	Temporal spatial, kinematics and kinetics: 3DGA Patient perceived comfort	3DP AFO significantly increased ankle dorsiflexion compared with shod, but not with Codivilla. 3DP AFO enabled faster walking compared to the Codivilla and shod only. 3DP AFO was rated as more comfortable and had higher perceived push than the Codivilla.	4
				Fibreglass reinforced polyamide				
Caravaggi 2024 [14]	10	Unilateral foot drop due to the compression of the lumbosacral spine (8 M, 2 F, 64.9 SD 11.4 years)	PDAFO	SLS	Shod only; Off‐the‐shelf leaf spring AFO (Codivilla)	Temporal spatial, kinematics and kinetics: 3DGA Patient perceived comfort Muscle activation: sEMG	3DP AFO effectively supported the foot during the swing phase by preventing passive ankle plantarflexion and reducing sagittal‐plane ROM. 3DP AFO enabled significantly faster walking speed and longer stride length compared to walking without an orthosis, but not compared with Codivilla. 3DP AFO was rated as significantly more comfortable and had higher perceived push than the Codivilla. Peak of sEMG signal of the gastrocnemius was different only in the Codivilla condition.	4
				Fibreglass reinforced polyamide				
Caravaggi 2025 [13]	8	Unilateral foot drop due to compression of the lumbosacral spine (3 F, 5 M, 62.0 SD 13.1 yrs)	PDAFO	SLS	Shod only; Off‐the‐shelf leaf spring AFO (Codivilla)	Temporal spatial, kinematics and kinetics: 3DGA	Reduced maximum normalised ankle power at push off. Walked faster with AFOs compared to shod only. Energy absorbed and released was small compared to the work produced at the ankle at push off.	4
				Fibreglass reinforced polyamide				
Cha 2017 [16]	1	Right side foot drop after embolectomy (F, 68 years)	Customised AFO	FDM	Traditionally made AFO	User satisfaction QUEST after 2 months Temporal spatial, ankle kinematics: 3DGA	The participant was more satisfied with 3DP AFO in terms of weight and ease of use. Temporal spatial parameters were similar; however, ankle dorsiflexion in swing was less with the 3D printed AFO compared to the traditional one.	4
				Polyurethane				
Chae 2020 [30]	1	Lumbar radiculopathy (F, 72 years)	NR	FDM polyurethane	No control	Stability User satisfaction QUEST 6MWT Ambulatory function: mEFAP	3DP AFO improved stability scores. Participant gave 3DP AFO a perfect QUEST score. 3DP AFO increased distance on 6MWT. 3DP AFO improved total mEFAP score.	4
Cho 2023 [17]	3	Right basal ganglia haemorrhage, multifocal scattered infarction, right putamen haemorrhage (M, 30,58,47 years)	NR	FDM polyester elastomer	Traditionally made AFO; barefoot; shod only	Temporal spatial, kinematics and kinetics: 3DGA Muscle strength User satisfaction: SUS and open ended questions FM‐L, BBS, and 6MWT Social participation	3DP AFO resulted in more symmetrical gait and increased step length, walking speed and stride length than other conditions. After 4‐week gait training with 3DP AFO, walking speed and step length increased over the baseline. There was no change in stride width and decreased symmetry. The participants were satisfied with 3DP AFO. Gait training with both 3DP AFO and traditional AFO led to improvements in physical impairments, activity limitations and reduction in depression with no improvement in social participation.	4
Choi 2017 [32]	8	Healthy (4 M, 4 F, 25.3 SD 4.5 years)	Hinged AFO	FDM	No control	Kinematics: 3DGA Ultrasound Musculoskeletal modelling	Increasing AFO stiffness increased peak ankle dorsiflexion moment and decreased peak knee extension and peak ankle dorsiflexion.	4
				PLA				
Creylman 2013 [18]	8	Unilateral drop foot due to dorsiflexor weakness from multiple conditions (m, 46.6 SD 12.5 years)	PLS	SLS	Traditionally made AFO; barefoot	Temporal spatial and kinematics and kinetics: 3DGA	No statistically significant differences in terms of temporal spatial gait parameters, ankle angle at initial contact and maximum ankle plantarflexion during swing. Significant differences were noted in the ankle range of motion.	4
				PA2201				
Dabnichki 2025 [33]	NR	Healthy	NR	SLS	No control	Patient perceived comfort Effectiveness from observation	Discomfort was observed around the calf connection. The 3DP AFO showed excessive stiffness. The usability was enhanced.	5
				PA12				
Deckers 2018 [19]	7	Trauma, Neuromuscular disorder and cerebral palsy (3 children, 4 adults)	Segmented AFO with posterior interchangeable calf strut	SLS	Traditionally made AFOs	Observation after 6 weeks.	No noticeable failure or wear with the traditionally manufactured AFOs after 6 weeks 5/7 SLS AFO broke during the 6‐week period, 1 SLS AFO showed signs of cracking and 1 did not fail.	4
				PA12				
Fu 2022 [20]	10	Unilateral stroke (8 M, 2 F, 54 SD 13 years)	Hinged AFO	FDM	Traditionally made anterior AFO; shod only	Fatigue: 10mWT User satisfaction: QUEST Plantar pressure	No significance difference in gait speed and cadence among the three groups. 3DP AFO and traditional AFO had comparable satisfaction. Compared to barefoot, 3DP and traditional AFOs significantly increased medial midfoot plantar pressures. 3DP AFO showed more medial weight bearing and symmetric contact area over the sole than the traditional AFO.	4
				PLA				
Funes‐Lora 2022 [31]	1	Healthy participant (M, 33 years)	Segmented AFO with posterior interchangeable calf strut	FDM	No control	Self‐reported satisfaction Durability: observation	No damage to 3DP AFO after 4 months of wear. No unwanted gait modifications. No discomfort experienced.	4
				Nylon 12				
Koller 2021 [21]	10	Chronic hemiparesis stroke (7 M, 3 F, 56.3 SD 9.02 years)	PDAFO	NR	Traditionally made hinged AFO	Temporal spatial, kinematics and kinetics: 3DGA	Significant increase in mean peak paretic plantar flexion moment with 3DP AFOs compared with traditional AFOs. Inconsistent results seen in biomechanical and walking performance parameters across the participants.	4
				Polycarbonate/carbon fibre				
Kumar 2023 [22]	3	Unilateral foot drop weight between 55 and 60 kg	Segmented AFO with posterior carbon fibre calf strut	FDM	NR	Temporal spatial, kinematics and kinetics: 3DGA	Improvement heel contact and loading response for the 3DP AFO condition. Excessive plantarflexion in the sagittal plane during the swing phase decreased for the 3DP AFO condition. Mean velocity increased for the 3DP AFO condition.	4
				ABS (calf and foot); nylon composite carbon fibre (strut)				
Li 2022 [35]	5	Able bodied (4 M, 1 F)	PDAFO with a quick release strut	NR	Identical 3DP AFO with the screw anchor mechanism	Quick release efficiency: Timed swapping tests	The quick release mechanism reduced strut swap time compared to screw‐anchor connections.	5
				NR				
Lin 2021 [23]	12	Post stroke (3 M, 9 F, 55.58 SD 5.9 years)	Novel designed energy‐storage AFO	FDM	Traditionally made anterior AFO; barefoot	Temporal spatial, kinematics and kinetics: 3DGA User satisfaction: QUEST Fatigue: 10mWT	Shank and ankle kinematics were closer to normative populations for the early loading response phase for both AFO subgroups compared to barefoot. 3DP AFO created a higher moment at 35%–60% of the gait cycle than the traditional AFO or barefoot. 3DP AFO gait velocity was significantly faster than the traditional AFO or barefoot. 3DP AFO had significantly higher satisfaction than the traditional AFO. There were no significant differences of fatigue between conditions.	4
				PLA (shank); nylon (joints)				
Liu 2019a [24]	8	Post stroke (4 M, 4 F, 57.25 SD 9.95 years)	NR	SLA	NR	Temporal spatial outcomes: gait analysis (sensors)	Velocity and stride length were significantly increased in the 3DP AFO condition. No significant difference in cadence, double limb support, step length ratio, swing phase ratio and stance phase ratio in the 3DP AFO condition.	4
				Somos NeXt				
Liu 2019b [25]	12	Post stroke (8 M, 4 F, 55.8 SD 9.2 years)	NR	Powder bed fusion	NR	Temporal spatial, kinematics and kinetics: gait analysis (sensors) Duration & dimension accuracy: observation Satisfaction and fit: self‐report and observation	Significant increases in velocity and stride length in the 3DP AFO condition. No significant difference in cadence, double limb support and step length ratio. No reported 3DP AFO breakage over 3 months, with no skin pressure sores. 3DP AFOs fit well, and compliance was good.	4
				Nylon 12				
Mavroidis 2011 [26]	1	Healthy	Rigid & flexible AFOs	SLA Accura 40 resin and DSM somos 9120 Epoxy Photopolymer	Prefabricated injection moulded polypropylene AFO, shod only	Temporal spatial, kinematics and kinetics: 3DGA Patient perceived fit	3DP AFOs provided a good fit to the participant's anatomy and were comparably to the prefabricated AFO during gait.	5
Schrank and stanhope 2013 [36]	2	Healthy participants (M 48 years; F 21 years)	PDAFO	SLS	No control	Patient perceived fit	Subjective evaluations of the full‐scale PDAFOs were positive.	4
				Nylon (DuraForm EX Natural plastic)				
Telfer 2012 [37]	1	Healthy participant (M 29 years)	Segmented AFO with posterior interchangeable calf strut	SLS	Shod only	Kinematics and kinetics: 3DGA	3DP AFO had distinct effects on ankle kinematics which could be varied by adjusting the stiffness.	5
				Nylon‐12 (PA2200)				
Vasiliauskaite 2020 [28]	1	Unilateral foot drop (M, 7 years)	PLS	NR	Traditionally made hinged AFO; shod only	Temporal spatial, kinematics and kinetics: 3DGA	Excessive ankle plantarflexion at initial contact, mean ankle angle in the end swing was reduced, and second rocker reversal was prevented by both AFO conditions compared to shod at baseline and after 6 weeks of wear. Only the traditional AFO decreased excessive ankle plantarflexion above MCID. For both AFO conditions, speed increased compared with shod gait. Peak ankle push‐off power increased with 3DP AFO compared with shod condition, but not in the control AFO condition at baseline. After 6 weeks of wear, peak ankle push‐off power increase was maintained in 3DP AFO and increased in the control AFO condition compared to shod.	4
				Nylon 12				
Vasiliauskaite 2021 [27]	6	Poly‐trauma, CMT, CP, clubfoot (3 M, 3 F, 23 SD 18 years)	Segmented AFO with posterior changeable carbon rod	SLS	Traditionally made PLS; shod only	Temporal spatial, kinematics and kinetics: 3DGA	For all conditions, the ankle range of motion and mean ankle velocity significantly decreased compared to shod. Step length significantly increased with 3DP AFO compared to shod, but not with control AFO. Inconsistent results across participants, so split into ‘good’ and ‘bad’ responders.	4
				Nylon 12 (footplate and calf)				
Wojciechowski 2022 [29]	12	CMT (6 M, 6 F, 11.2 SD 3.6 years)	Hinged AFO; Solid + supramalleolar insert; redesigned AFO	FDM	Traditionally made hinged AFO; solid + supramalleolar insert AFO; shod only	Temporal spatial, kinematics and kinetics: 3DGA User satisfaction: CSD‐OPUS Plantar pressure	3DP replica AFOs produced equivalent biomechanical outcomes and self‐reported satisfaction scores to traditional handmade AFOs made by experienced paediatric orthotists. Redesigned AFOs were significantly lighter and provided a normalised maximum ankle dorsiflexor moment in loading response. 3DP replica AFOs improved foot position at initial contact and during loading responses and significantly reduced pressure beneath the whole foot, rearfoot and forefoot compared to shoes only.	4
				Nylon 12				

Abbreviations: 3DGA: three‐dimensional gait analysis, 3DP: three‐dimensional printing, 6MWT: 6‐min walk test, 10mWT: 10‐m walk test, ABS: acrylonitrile butadiene styrene, AFO: ankle foot orthosis, BBS: Berg balance scale, CMT: Charcot‐Marie‐Tooth disease, CP: cerebral palsy, CSD‐OPUS: client satisfaction with device of the orthotic and prosthetic users survey, F: female, FDM: fused deposition modelling, FM‐L: Fugl–Meyer lower extremity, M: male, MCID: minimal clinically important difference, mEFAP: modified Emory functional ambulation profile, NR: not reported, OCEBM: Oxford centre for evidence‐based medicine levels of evidence, PDAFO: passive dynamic ankle foot orthosis, PLA: polylactic acid, PLS: posterior leaf spring AFO, QUEST: Quebec user evaluation of satisfaction with assistive technology, sEMG: surface electromyography, SLA: stereolithography, SLS: selective laser sintering, SUS: system usability scale.

^a^ The paper's OCEBM level was downgraded one level as the quality of the evidence is very low.

### 3.3 3D Printed AFOs Compared to Traditional or Prefabricated AFOs

Seventeen out of the 28 papers (60.7%) reported and compared outcomes of 3D printed AFOs with other AFOs such as traditionally made or prefabricated AFOs [10, 11, 13, 14, 15, 16, 17, 18, 19, 20, 21, 23, 26, 27, 28, 29, 35]. Walking speed and step/stride length were the only outcomes reported with significant increases compared to traditionally made AFOs by only two studies [23, 27], whereas others were generally comparable. Cho et al. revealed an increase of walking speed, step length and the functional test without statistical significance [17]. Wojciechowski et al. described a significant increase in maximum ankle plantarflexion at push off, even though maximum ankle dorsiflexion in swing and ankle dorsiflexion at initial contact were decreased, as well as a significant decrease of the total peak pressure, when comparing the novel 3D redesigned AFOs with traditional AFOs [29]. Moreover, two studies observed significant increases in ankle moment, specifically peak paretic plantarflexor moment in stance and maximum ankle dorsiflexor moment in loading responses [21, 29]. However, the 3D printed replicas that shared the same design and shape as the traditional AFOs were shown to have no significant differences in all outcomes compared to traditional methodologies [29]. In addition, Vasiliauskaite et al. reported an increase of the peak ankle push‐off power [27, 28] and Arch mentioned an increase of peak plantarflexion moment without statistical significance [11]. In addition, two studies tested both immediate effects and the effect at a follow‐up of 4 weeks [17] and 6 weeks [28].

Four papers reported an improvement of patient‐perceived comfort/satisfaction [14, 15, 16, 23], whereas two reported comparable comfort/satisfaction results between 3D printed AFOs and traditionally made AFOs [20, 29]. One study highlighted the discomfort caused by the difficulty of wearing the 3D printed AFO by the participants themselves, even though the 3D printed AFOs were better in terms of weight, fit, aesthetics and safety [17], whereas another study pointed out a reduction of the time required for the patient fitting [19]. More outcome details can be found in Supporting Information S2: Additional file 3.

### 3.4 3D Printed AFOs Compared to Shod or Barefoot Only

There were comparisons reported between 3D printed AFOs and non‐AFO situations such as shod only or barefoot. Seven out of 19 studies highlighted significant increases in terms of walking speed and/or step/stride length [14, 18, 23, 24, 25, 27, 28]. Ankle kinematics was reported under different conditions and showed inconsistent results. Significant reductions in ankle plantarflexion angles during swing/stance and at push‐off were reported by Caravaggi and Telfer [14, 37]. Some studies highlighted significant increases in ankle dorsiflexion in swing [23] and max plantarflexion at push off [29]. However, interestingly, conflicting results were reported in two similar studies conducted by Vasiliauskaite et al., of which maximum ankle dorsiflexion in stance was significantly increased in one but decreased in the other [27, 28], which might be due to the variability in tested participants and different intended purposes of the AFOs. Six out of seven papers that tested ankle moment/power showed improvements [11, 12, 13, 22, 23, 28, 29]. Two articles found significant increases regarding peak ankle push‐off power, maximum ankle dorsiflexor moment in loading response and maximum ankle plantarflexor moment [28, 29], whereas one mentioned a significant reduction on peak ankle internal plantarflexion moment [37]. More outcome details can be found in Supporting Information S2: Additional file 4.

### 3.5 Study Quality

Study quality of each individual paper was assessed by QualSyst. In total, ten out of the 28 included papers (35.7%) were rated as excellent of which six were papers published after 2018 [13, 14, 20, 21, 28, 29], and four (14.3%) were rated as good [15, 23, 27, 36]. It is noteworthy that papers published prior to 2018 exhibited a higher proportion of excellent quality (44.4%) compared to those published more recently (31.6%). The details of the questions and answers were listed in Table 2.

**TABLE 2:** Quality of the included studies as assessed by QualSyst.

	Q1	Q2	Q3	Q4	Q5	Q6	Q7	Q8	Q9	Q10	Q11	Q12	Q13	Q14	Overall
Abdalsadah 2021	Partial	No	No	Partial	N/A	N/A	N/A	No	No	No	No	No	No	No	9.1%
Arch 2015	Yes	Yes	Yes	Yes	Partial	N/A	N/A	Yes	N/A	Partial	Yes	N/A	Yes	Yes	90.0%
Arch 2016	Yes	Yes	Yes	Yes	Partial	N/A	N/A	Yes	N/A	Partial	N/A	N/A	Yes	Yes	88.9%
Banga 2022	No	No	No	No	N/A	N/A	N/A	No	Partial	No	No	No	No	No	4.5%
Caravaggi 2022	Partial	Yes	Yes	Yes	Partial	N/A	N/A	Yes	No	N/A	Yes	Partial	Yes	Yes	77.3%
Caravaggi 2024	Yes	Yes	Yes	Yes	Partial	N/A	N/A	Yes	Yes	Yes	Yes	Partial	Yes	Yes	91.7%
Caravaggi 2025	Yes	Yes	Yes	Yes	No	N/A	N/A	Yes	N/A	Yes	Partial	N/A	Yes	Yes	85.0%
Cha 2017	Partial	Partial	N/A	Partial	N/A	N/A	N/A	Yes	N/A	Partial	N/A	N/A	Yes	Yes	71.4%
Chae 2020	Partial	Partial	N/A	Yes	N/A	N/A	N/A	Yes	N/A	N/A	N/A	N/A	Partial	Partial	66.7%
Cho 2023	Yes	Yes	No	Yes	Partial	N/A	N/A	Yes	Partial	Partial	Yes	No	Yes	Yes	70.8%
Choi 2017	Yes	Yes	Yes	Yes	Partial	N/A	N/A	Yes	Yes	Yes	Yes	N/A	Yes	Yes	95.5%
Creylman 2013	Yes	Yes	Yes	Yes	No	N/A	N/A	Yes	Yes	Yes	Yes	N/A	Yes	Yes	90.9%
Dabnichki 2025	No	No	No	No	N/A	N/A	N/A	Partial	N/A	N/A	N/A	N/A	Partial	No	14.3%
Deckers 2018	No	Partial	Partial	Partial	Partial	N/A	N/A	N/A	N/A	N/A	N/A	N/A	Partial	Yes	50.0%
Fu 2022	Yes	Yes	Yes	Yes	Partial	N/A	N/A	Yes	Partial	Yes	Yes	Partial	Yes	Yes	87.5%
Funes‐Lora 2022	Partial	Partial	N/A	Yes	N/A	N/A	N/A	No	N/A	N/A	N/A	N/A	Partial	Yes	58.3%
Koller 2021	Yes	Yes	Yes	Yes	No	N/A	N/A	Yes	Yes	Yes	Yes	Partial	Yes	Yes	87.5%
Kumar 2023	Partial	Partial	Partial	Partial	N/A	N/A	N/A	Yes	Partial	Partial	Partial	No	Yes	Partial	54.5%
Li 2022	Partial	Partial	Partial	No	N/A	N/A	N/A	Yes	Partial	Partial	Yes	N/A	Yes	Partial	60.0%
Lin 2021	Yes	Yes	Yes	Yes	No	N/A	N/A	Yes	Yes	Yes	Partial	Partial	Yes	Yes	83.3%
Liu 2019a	Partial	Partial	Yes	Yes	No	N/A	N/A	Partial	Yes	Yes	Yes	Partial	Partial	No	62.5%
Liu 2019b	Partial	No	Yes	Yes	No	N/A	N/A	No	Yes	Yes	Yes	Partial	No	No	50.0%
Mavroidis 2011	Partial	Partial	N/A	No	N/A	N/A	N/A	Yes	N/A	N/A	Partial	N/A	Yes	Yes	64.3%
Schrank and stanhope 2013	Yes	Yes	Partial	Partial	N/A	N/A	N/A	Partial	N/A	Yes	Yes	N/A	Yes	Yes	83.3%
Telfer 2012	Partial	Partial	Partial	Partial	N/A	N/A	N/A	Yes	N/A	Yes	Partial	N/A	Yes	Yes	72.2%
Vasiliauskaite 2020	Yes	Yes	Partial	Yes	No	N/A	N/A	Yes	N/A	Yes	Yes	N/A	Yes	Yes	85.0%
Vasiliauskaite 2021	Yes	Yes	Yes	Yes	No	N/A	N/A	Yes	Yes	Yes	Partial	Partial	Yes	Yes	83.3%
Wojciechowski 2022	Yes	Yes	Yes	Yes	Partial	N/A	N/A	Yes	Yes	Yes	Yes	Partial	Yes	Yes	91.7%

*Note:* Q1: Question/objective sufficiently described? Q2: Study design evident and appropriate? Q3: Method of subject/comparison group selection or source of information/input variables described and appropriate? Q4: Subject (and comparison group, if applicable) characteristics sufficiently described? Q5: If interventional and random allocation was possible, was it described? Q6: If interventional and blinding of investigators was possible, was it reported? Q7: If interventional and blinding of subjects was possible, was it reported? Q8: Outcome and (if applicable) exposure measure(s) well defined and robust to measurement/misclassification bias? Means of assessment reported? Q9: Sample size appropriate? Q10: Analytic methods described/justified and appropriate? Q11: Some estimate of variance is reported for the main results? Q12: Controlled for confounding? Q13: Results reported in sufficient detail? Q14: Conclusions supported by the results?.

## 4 Discussion

This updated systematic review has highlighted the rapid growing trend of using additive manufacturing to produce AFOs. There were more included papers published since 2018 than papers included in the previous review of which the publication period was from 1985 to 2018. The findings of this review revealed that 3D printed novel designed AFOs have had potential benefits for improving biomechanical outcomes compared to traditional or prefabricated AFOs. The 3D printing techniques utilised varied considerably, with SLS predominating in studies published prior to 2018, whereas FDM has dominated in more recent literature. Nylon 12 has remained as the most popular 3D printing material, and most of the studies 3D printed passive dynamic AFOs or PLS. The sample sizes of the included studies were all smaller than 12 and there were not enough data from normative tests. Intriguingly, papers published before 2018 were more likely to be a proof of concept (44.4%) rather than clinical studies, which is opposite to the studies published more recently (15.8%).

More than half of the included studies (62.1%) compared 3D printed AFOs to other AFOs. Walking ability was mostly improved by 3D printed AFOs as well as device comfort and satisfaction compared to traditionally made or prefabricated AFOs with similar design features. Almost all included papers compared novel designed AFOs to traditional AFO shapes, whereas Wojciechowski et al. compared the 3D printed replica to the traditional AFOs that shared the exact same design, showing the 3D printed replica was equivalent to the traditional AFOs in terms of biomechanical outcomes and self‐reported satisfaction scores amongst children with CMT [29]. It might indicate that the 3D printed AFOs with traditional designs were expected to perform the same as the traditional ones so that it is more meaningful to test novel designs rather than traditional designs. Amongst papers that compared with shod or barefoot conditions, many reported significant improvements in walking speed and step length, ankle dorsiflexion in swing and secondary improvements of ankle moment and push‐off power. However, the reported outcomes were selective and inconsistent. In addition, it should be noted that the evidence synthesis of the comparisons regarding kinematic and/or kinetic parameters across studies can be unnecessary due to the different goals of the prescribed AFOs, desired shank to vertical angle and the varieties of the participants' gait patterns. Therefore, it is essential to state the intended goal of the AFO to ensure that the outcomes are clinically meaningful. It would also be beneficial to the research community to establish an agreement for how and what to evaluate and report in terms of spatiotemporal, kinematic, kinetic parameters satisfaction score, functional tests and long‐term usage.

Much like the earlier review, 3D printing materials and methods varied across the included studies. The most popular material was Nylon 12, followed by PLA. Surprisingly, no included study used Nylon 11, despite the material being prevalent in the industry and demonstrated appropriate properties in terms of mechanical damping and deformation [38]. The 3D printing methods included SLS, SLA, power bed fusion and FDM. SLS was more popular than FDM within papers prior to 2018, whereas FDM was the dominant method amongst publications after 2018, although anecdotally SLS was increasingly widespread in industry. This may be attributed to the rapid growth and widespread adoption of FDM within the 3D printing market, which has increased its accessibility. Moreover, none of the papers have mentioned the post‐processing protocols and there is still limited information about the lifespan of the 3D printed AFOs apart from Funes‐lora et al. reporting no damage to the AFO after 4 months of wear [31], whereas Deckers et al. mentioned only one out of six SLS AFOs survived after 6 weeks of wear [19]. In the future, more information regarding post‐processing should be reported, and further research should be encouraged towards how the post‐processing component would affect the mechanical properties and wearability features of the AFO. Especially in the paediatric setting, one of the challenges faced today is the limitation of adjusting shapes after AFOs have been 3D printed. In contrast, traditional handmade thermoplastics allows for spot heating which enables the same device to be used for growth and pressure alleviation over bony prominences. There are also health and safety concerns of the traditional manufacturing method. Studies of the Orthotic and Prosthetic Workforce in Australia in 2019 depicted that the prevalence of professionals obtaining a workplace injury was at a rate f 25.9% compared with other allied health professionals who had a prevalence at less than 1.5% [39]. This was largely due to many departments having an industrial workshop setup with workers frequently exposed to volatile noise, repetitive manual tasks and exposure to dust particles [39]. However, this could be potentially solved by delicate 3D scanning and 3D modelling programmes for achieving the desired shape and better working environment.

Excitingly, more and more research has been undertaken looking at patient specific outcome measures when comparing traditional to 3D printed AFOs. It would be beneficial to conduct studies further investigating specific condition characteristics such as cerebral palsy within a specific Gross Motor Function Classification System (GMFCS) level, weight range, functional supports utilised, gait characteristics and functional goals. Furthermore, although 3D technologies can unlock novel AFO designs and all papers have claimed supportive results, it is critical that the designs should be rigorously tested including fatigue and wear characteristics as well as incorporating consistent designs, shank to vertical angles, footwear and tested on patients before widespread adoption and use in an orthotics setting.

There are several limitations of this review. First, the review only focused on the feasibility of 3D printed AFOs based on functional tests in human populations. Any studies assessing AFOs using mechanical or computational tests were not included, which limits the available evidence base to support the feasibility of 3D printed AFOs. Second, studies where only a minor part of the AFO was 3D printed were excluded, which omits studies supporting a potential viable strategy for the use of this technology. Third, the search strategy may have missed some studies due to poor reporting, such as those that did not use conventional 3D printing terms in the text or did not provide a clear statement on the use of 3D printing. Forth, we did not extract details regarding AFO designs and if the information provided was sufficient for replication. To strengthen this review and others in the future, quality assessment tools could rate whether sufficient description of AFO designs for reproduction was provided.

## 5 Conclusion

The number of studies using additive manufacturing in AFO fabrication has rapidly increased in the recent past. Novel designed 3D printed AFOs might have potential benefits over traditional designs and 3D printed AFOs have been generally proved to be comparable to traditional ones. Walking speed and step length of people wearing 3D printed AFOs were the two elements that were most improved when compared to other AFO and non‐AFO conditions. 3D printing methods and materials still varied markedly. FDM and SLS were both the predominant 3D printing methods, although SLS was more frequently utilised in studies published prior to 2018. Nylon 12 remained the most tested material. We recommend further research in specific populations such as those with cerebral palsy within a specific GMFCS level, longitudinal clinical trials and testing in a community environment. Establishing a standard for AFO evaluation and reporting would also enable valuable comparisons between studies.

## Author Contributions

**Joyce Z. Wang:** conceptualisation, data curation, formal analysis, methodology, writing – original draft, writing – review and editing. **Elizabeth A. Wojciechowski:** conceptualisation, writing – review and editing. **Thomas Paine:** writing – review and editing, validation. **Joshua Burns:** conceptualisation, funding acquisition, resources, writing – review and editing. **Tegan L. Cheng:** conceptualisation, funding acquisition, resources, data curation, writing – review and editing.

## Funding

J.B. receives research funding from the Australian Government (Grants NHMRC #20150180, MRFF #2022/MRF2015970), National Institutes of Health (Grants #U01NS109403, #U54NS065712), Muscular Dystrophy Association (Grants #1060929) and Charcot‐Marie‐Tooth Association. T.L.C. receives research funding from the Australian NHMRC (Grant #1194930). The EPIC Lab is supported by Hyundai Help for Kids Australia.

## Ethics Statement

The authors have nothing to report.

## Consent

The authors have nothing to report.

## Conflicts of Interest

The authors declare no conflicts of interest.

## Supporting information


Supporting Information S1

Supporting Information S2

**Table S1:** Search strategy for medline, modified for other databases.

## Data Availability

The authors have nothing to report.
