# Supplementary material for: Feasibility of Designing, Manufacturing and Delivering 3D Printed Ankle‐Foot Orthoses: An Updated Systematic Review
Source: J Foot Ankle Res. 2025 Dec 9;18(4):e70097. doi: 10.1002/jfa2.70097 (PMC12687059; doi:10.1002/jfa2.70097)
Supplement: Supplementary file 2 — Supporting Information S2 [file JFA2-18-e70097-s002.docx]

**Additional file 3. Outcomes of 3DP-AFO compared with that of other AFOs.**

| **Reference** | **Wearing period before testing** | **Outcomes** | | | | | | |
| --- | --- | --- | --- | --- | --- | --- | --- | --- |
|  |  | **Walking velocity** | **Step/stride length** | **Ankle kinematics** | **Functional test** | **Ankle power / moment** | **comfort/**  **satisfaction** | **Plantar parameters** |
| Abdalsadah 2021 | NR | NR | NR | NR | NR | NR | NR | NR |
| Arch 2016 | Immediate effect | NR | NR | Improved | NR | Peak plantarflexion moment: ↑ | NR | NR |
| Caravaggi 2022 | Immediate effect | ↑ | NR | Dorsiflexion in swing: - | NR | NR | comfort: ↑ | NR |
| Caravaggi 2024 | Immediate effect | - | - | NR | NR | NR | comfort: ↑ | NR |
| Caravaggi 2025 | Immediate effect | - | NR | NR | NR | - | NR | NR |
| Cha 2017 | 2 months | - | - | Dorsiflexion in swing: ↓ | NR | NR | Satisfaction in weight and easy of use: ↑ | NR |
| Cho 2023 | Immediate effect | ↑ | ↑ | NR | ↑ | NR | Inconsistent | NR |
|  | 4 weeks |  |  |  |  |  |  |  |
| Creylman 2013 | At least half an hour | NR | - | Ankle range of motion: ↓* | NR | NR | NR | NR |
| Deckers 2018 | 6 weeks | NR | NR | NR | NR | NR | Fitting time: ↓ | NR |
| Fu 2022 | NR | - | NR | NR | NR | NR | Satisfaction: - | Medial midfoot contact area and peak pressure of unaffected limb: ↑* |
| Koller 2021 | Immediate effect | Inconsistent | Inconsistent | Inconsistent | Inconsistent | Peak paretic plantarflexion moment: ↑* | NR | NR |
| Li 2022 | NR | NR | NR | NR | NR | NR | NR | NR |
| Lin 2021 | NR | ↑* | Stride length: ↑* | - | - | ↑ | Satisfaction: ↑ | NR |
| Mavroidis 2011 | NR | NR | NR | Ankle range of motion during controlled dorsiflexion: ↓ | NR | - | NR | NR |
| Vasiliauskaite 2020 | Immediate effect and | NR | NR | Plantarflexion end swing and initial contact: ↓ | NR | Peak ankle push-off power: ↑ | NR | NR |
|  | 6 weeks | NR | NR | Plantarflexion end swing and initial contact: ↓ | NR | Peak ankle push-off power: ↑ | NR | NR |
| Vasiliauskaite 2021 | Immediate effect | ↑** | ↑** | - | NR | ↑ | NR | NR |
| Wojciechowski 2022 | Immediate effect | Replica: - | Replica: - | Replica: - | NR | Replica: - | Replica: - | Replica: - |
|  |  | Redesign: - | Redesign: - | Redesign: Max ankle dorsiflexion in swing: ↓*  Ankle dorsiflexion at initial contact↓*  Max ankle plantarflexion at push off ↑* |  | Redesign: Max ankle dorsiflexor moment in loading response ↑* | Redesign: - | Redesign: Total peak pressure ↓* |
| NR: not reported.  -: no significant difference or was comparable.  *: reported statistically significant.  **: statistically significant but not exceed minimal clinically important difference (MCID). | | | | | | | | |

**Additional file 4. Outcomes of 3DP-AFO compared with that of barefoot or shod only conditions**

| **Reference** | **Wearing period before testing** | **Outcomes** | | | | | |
| --- | --- | --- | --- | --- | --- | --- | --- |
|  |  | **Walking velocity** | **Step/stride length** | **Ankle kinematics** | **Functional test** | **Ankle power/moment** | **Plantar parameters** |
| Banga 2022 | NR | NR | Inconsistent | Inconsistent | Inconsistent | NR | NR |
| Arch 2015 | Immediate effect | NR | NR | Peak dorsiflexion in stance: ↓ |  | Peak plantarflexion moment: ↑ | NR |
| Arch 2016 | Immediate effect | NR | NR | Dorsiflexion: ↓ | NR | Peak plantarflexion moment: ↑ | NR |
| Caravaggi 2022 | Immediate effect | ↑ | NR | Dorsiflexion in swing: ↑ | NR | NR | NR |
| Caravaggi 2024 | Immediate effect | ↑* | Stride length: ↑* | Plantarflexion in swing/stance: ↓* | NR | NR | NR |
| Caravaggi 2025 | Immediate effect | ↑ | NR | NR | NR | Max normalised ankle power at push-off: ↑ | NR |
| Cha 2017 | 2 months | ↑ | ↑ | Dorsiflexion in swing: ↑ | NR | NR | NR |
| Cho 2023 | Immediate effect | ↑ | ↑ | NR | NR | NR | NR |
|  | 4 weeks | NR | NR |  |  |  |  |
| Creylman 2013 | At least half an hour | NR | ↑* | Maximum ankle plantarflexion during swing and ankle range of motion: ↓* | NR | NR | NR |
| Fu 2022 | NR | - | - | NR | NR | NR | Medial midfoot contact area and peak pressure of affected limb: ↑* |
| Kumar 2023 | 7 days | ↑ | NR | Plantarflexion in swing: ↓ | NR | Ankle moment at preswing: ↑ | NR |
| Lin 2021 | NR | ↑* | Stride length: ↑* | Dorsiflexion in swing: ↑* | - | ↑ | NR |
| Liu 2019a | Immediate effect | ↑* | Stride length: ↑* | NR | NR | NR | NR |
| Liu 2019b | Immediate effect | ↑* | Stride length: ↑* | NR | NR | NR | NR |
| Mavroidis 2011 | NR | NR | NR | Peak plantarflexion at in swing: ↓ | NR | - | NR |
| Telfer 2012 | NR | NR | NR | Peak plantarflexion angle at the start of stance and Plantarflexion angle at push off: ↓* | NR | Peak ankle internal plantarflexion moment: ↓* | NR |
| Vasiliauskaite 2020 | Immediate effect | ↑** | ↑** | Plantarflexor in swing: ↓  Max ankle dorsiflex in stance: ↑** | NR | Peak ankle push-off power: ↑** | NR |
|  | 6 weeks | ↑ | ↑ |  |  |  |  |
| Vasiliauskaite 2021 | Immediate effect | ↑ | ↑** | Max ankle dorsiflex in stance: ↓* | NR | NR | NR |
| Wojciechowski 2022 | Immediate effect | Replica and Redesign: - | Replica and Redesign: - | Replica and Redesign:  Max ankle plantarflexion at push off ↑* | NR | Replica: Max ankle dorsiflexor moment in loading response ↑*  Max ankle plantarflexor moment ↑* | Replica and Redesign: peak pressure beneath the rearfoot ↓*  Mean pressure beneath the whole foot, rearfoot and forefoot ↓*  Total pressure time integral ↓* |
|  |  |  |  |  |  | Redesign: Max ankle dorsiflexor moment in loading response ↑* |  |
| NR: not reported.  -: no significant difference or comparable.  *: reported statistically significant.  **: statistically significant and exceed minimal clinically important difference (MCID). | | | | | | | |
