# Supplementary material for: Feasibility of Designing, Manufacturing and Delivering 3D Printed Ankle‐Foot Orthoses: An Updated Systematic Review
Source: J Foot Ankle Res. 2025 Dec 9;18(4):e70097. doi: 10.1002/jfa2.70097 (PMC12687059; doi:10.1002/jfa2.70097)
Supplement: Supplementary file 3 — Table S1: Search strategy for medline, modified for other databases. [file JFA2-18-e70097-s001.docx]

**Additional file 1.** Search strategy for Medline, modified for other databases.

| **#** | **Search Statement** |
| --- | --- |
| 1 | Printing, Three-Dimensional/ |
| 2 | 3d print*.mp. |
| 3 | three dimensional print*.mp. |
| 4 | additive manufactur*.mp. |
| 5 | rapid prototyp*.mp. |
| 6 | additive fabricat*.mp. |
| 7 | additive process*.mp. |
| 8 | additive technique*.mp. |
| 9 | freeform fabricat*.mp. |
| 10 | solid freeform fabricat*.mp. |
| 11 | selective laser sinter*.mp. |
| 12 | sterolithography.mp. |
| 13 | fusion deposition model*.mp. |
| 14 | laminated object manufactur*.mp. |
| 15 | selective laser melt*.mp. |
| 16 | additive layer manufactur*.mp. |
| 17 | layer manufactur*.mp. |
| 18 | 1 or 2 or 3 or 4 or 5 or 6 or 7 or 8 or 9 or 10 or 11 or 12 or 13 or 14 or 15 or 16 or 17 |
| 19 | ankle foot ortho*.mp. |
| 20 | AFO*.tw. |
| 21 | exp animals/ not humans.sh. |
| 22 | 19 or 20 |
| 23 | 22 and 18 |
| 24 | 23 not 21 |
| 25 | limit 24 to (yr="2018 -Current" and english) |
